# Different effects on the tonus of colon and ileum isolated from mouse by resin glycoside (pharbitin) of Pharbitidis Semen

**DOI:** 10.1186/s12906-022-03570-9

**Published:** 2022-03-22

**Authors:** Jian-Rong Zhou, Naofumi Tokutomi, Yuusuke Satou, Shin Yasuda, Hideki Kinoshita, Toshihiro Nohara, Kazumi Yokomizo, Masateru Ono

**Affiliations:** 1 Department of Presymptomatic Medical Pharmacology, Faculty of Pharmaceutical Sciences, Sojo University, 4-22-1 Ikeda, Nishi-ku, Kumamoto, 860-0082 Japan; 2 Department of Bioscience, School of Agriculture, Tokai University, Kumamoto, 862-8652 Japan

**Keywords:** Resin glycoside, Pharbitin, Pharbitic acid, Pharbitidis semen, *Pharbitis nil*, Purgative, Colon tonus, Colon peristalsis, Ileum

## Abstract

**Background:**

Pharbitidis Semen (the seeds of *Pharbitis nil*), traditionally used as a purgative in Japan, China and Korea, contains a resin glycoside fraction named pharbitin, which is known as a purgative ingredient. Due to the complex nature of pharbitin, little is known about either the action on intestinal tension caused by resin glycoside itself or by its components.

**Methods:**

In this study, we investigated the effects of pharbitin, the glycosidic acid fraction (pharbitic acid) and the aglycone fraction (phar-genin) generated from pharbitin on peristalsis of colon and ileum isolated from mice with the Magnus method.

**Results:**

We demonstrated that pharbitin (3–30 μg/mL) concentration-dependently increased tonus of mice colon via acetylcholine receptors, its components phar-genin (1.27–12.7 μg/mL) and pharbitic acid (10–1000 μg/mL) also had the increment on colon tonus. On the other hand, ileum tension decreased in the presence of pharbitin.

**Conclusions:**

The effects of resin glycoside of Pharbitidis Semen on colon tonus are different with those on ileum tonus isolated from mice. In the next step it is necessary to investigate details of its pharmacological mechanism.

## Background

**Fig. 1:**
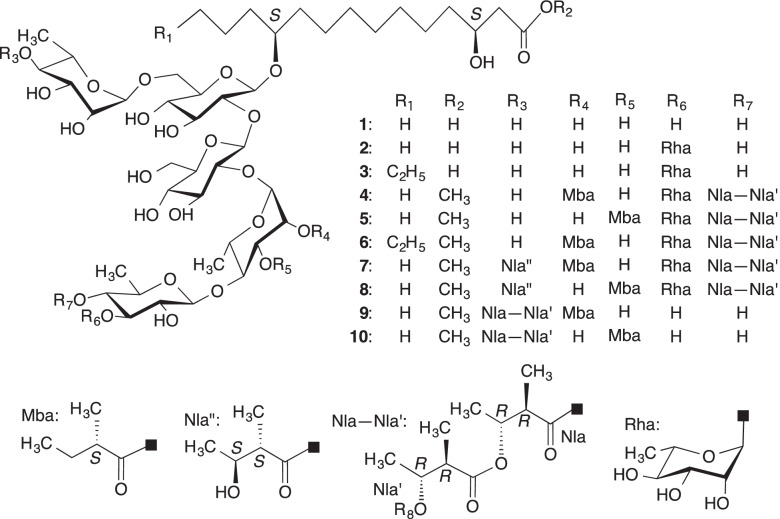
Structures of compounds 1–10 [1, 6]. Structures of component glycosidic acids (1–3) of pharbitin and acylated glycosidic acid methyl esters (4–10) generated from pharbitin by treatment with indium (III) chloride in MeOH

The resin glycosides were roughly classified into an acylated glycosidic acid with macrolactone-group called jalapin and an acylated glycosidic acid with free carboxylic acid-group denoted convolvulin [1]. Pharbitidis Semen, the seeds of *Pharbitis nil* (L.) Choisy has been used as a purgative in Japan, China and Korea [2, 3]. It has also been reported for having laxative properties in both rats and mice [4]. The first chemical investigation by Asahina in 1919 reported that alkaline hydrolysis of the crude resin glycoside named pharbitin gave three component organic acids and one glycosidic acid named pharbitic acid [5]. In 1990, three component glycosidic acids (1–3) generated by alkaline hydrolysis of pharbitin were reported (Fig. 1), and two hydroxyfatty acids, 3*S*,11*S*-dihydroxytetradecanoic acid and 3*S*,11*S*-dihydroxyhexadecanoic acid produced by acidic hydrolysis of the glycosidic acid fraction of pharbitin were isolated as methyl esters by Ono et al. [7]. Further, Ono et al. succeeded in isolation of seven acylated glycosidic acids as methyl esters (4–10) from pharbitin (Fig. 1) [6], and reported that a part of pharbitin was considered to be a mixture of free carboxylic acid forms corresponding to 4–10 [6].

Resin glycosides are characteristic ingredients of certain laxative crude drugs such as Mexican Scammony Radix, Orizabae Tuber, and Jalapae Tuber, all of which originate from Convolvulaceae plants [1]. A resin glycoside of jalapin type from *Ipomoea tricolor*, provoked spontaneous contractions of guinea pig ileum [8], however, those from *I. tyrianthina* resulted in relaxant effects on spontaneous contractions in isolated rat ileum [9]. The convolvulin type isolated from the Convolvulaceae family induced peristalsis in the small intestine [10], and that from *I. tyrianthina* did not inhibit significantly contractions on rat ileum and colon [11]. Furthermore, resin glycosides from Pharbitidis Semen have been reported to be responsible for laxative effect in MgSO_4_-induced constipation of rat [12]. Thus, the effects on intestinal motility by resin glycoside are different, it can be due to complex of glycoside. Hitherto, little is known about either the action on intestinal tension caused by the resin glycoside itself or by its components, and whether to increase the tonus of colon and ileum as well. Therefore, in this study, we extracted pharbitin from Pharbitidis Semen, and prepared its component glycosidic acid fraction (pharbitic acid) and aglycone fraction (phar-genin), then investigated their effects on peristalsis of ileum and colon isolated from mice with Magnus method.

## Methods

### Plant material

The seeds of *Pharbitis nil* (Lot. No. 00301800) was purchased in October 2018, from Tochimoto Tenkaido Co., Ltd., a commercial outlet of traditional medicines in Osaka, japan, and identified by emeritus Prof. Toshihiro Nohara, Faculty of Medical and Pharmaceutical Sciences, Kumamoto University. A voucher specimen has been deposited at the laboratory of Natural Products Chemistry, School of Agriculture, Tokai University.

### Extraction and preparation of pharbitin

The crushed seeds of *Pharbitis nil* (1.00 kg) were extracted with MeOH (1.00 L) at room temperature by standing still for 2 days, and the residue was re-extracted with MeOH (1.00 L) at room temperature by standing still for 2 days. The extract fluids were combined and the solvent was removed in vacuo to yield a MeOH extract (95.82 g). The MeOH extract was partitioned between 98% MeOH (550 mL) and hexane (150 mL × 3) to afford a 98% MeOH-soluble fraction (fr.) (70.96 g). The 98% MeOH-soluble fr. was applied to Diaion HP20 (Mitsubishi Chemical Industries) column chromatography eluted with H_2_O-MeOH (H_2_O, 70% MeOH, 100% MeOH) and acetone to yield 100% MeOH eluate (30.94 g, pharbitin).

### Preparation of pharbitic acid and phar-genin

Pharbitic acid and phar-genin were prepared as previously reported method [7]. Pharbitin (15.81 g) was suspended in 1% aqueous K_2_CO_3_ (100 mL) and heated at 95 °C for 1.5 h. After cooling, the reaction mixture was adjusted to pH 4 with 1 M HCl and shaken with ether (100 mL × 3). The aqueous layer was chromatographed over Diaion HP20 column (48 mm i.d. × 400 mm) eluted with H_2_O and acetone to afford acetone eluate (8.56 g, pharbitic acid).

Pharbitic acid (7.75 g) in 1 M HCl (5 mL) was heated at 95 °C for 1 h. The reaction mixture was extracted with ether (10 mL × 3). The ether extract was dried over MgSO_4_ and concentrated in vacuo to afford an aglycone fr. (phar-genin).

### Animals

The study was submitted to, and approved by the Ethics Committee of Sojo University (No. 2020-P-002). All experiments were conducted in strict accordance with the Guidelines of the Japanese Pharmacological Society for the Care and Use of Laboratory Animals. Twenty Male ddy mice, 6 ~ 8 weeks old, were obtained from Japan SLC (Hamamatsu, Japan). The animals were housed under a controlled room temperature (24.5–25.0 °C) at 60 ± 10% humidity under a 12/12 h light/dark cycle. Food pellets and tap water were provided ad libitum.

### Contractions of mice isolated colon and ileum by Magnus method

Mice were sacrificed by exposure to isoflurane (0.1 mL), and ileum and colon were dissected out and immediately immersed in Tyrode’s solution. Mouse ileum or colon was cut into rings (1–2 mm in length), placed in Magnus tube (20 mL; Medical Kishimoto, Kyoto, Japan) filled with Tyrode’s solution, maintained at 37 °C and bubbled with 95% O_2_ / 5% CO_2_ [13]. Then, each intestine ring was suspended by two stainless steel clips placed through the lumen. One clip was anchored to the bottom of the Magnus tube while the other was connected to an isometric transducer (UM-203; Medical Kishimoto), which was coupled to a dual-channel chart recorder (PowerLab 2/25; ADInstruments, Tokyo, Japan) for measurement of the isometric tension change. Each ring was stretched to a resting tension of about 4.9 mN and allowed to equilibrate for 5 min. Pharbitin or its components was cumulatively added to the Magnus tube at the corresponding concentrations. Amplitudes and areas of contraction were calculated from the polygraph tracing using LabChart® software. The peristalsis effects were determined by comparing amplitudes and areas under the curve inscribed by amplitude and frequency of colon and ileum contractions before and after the application of resin glycoside.

Pharbitin and pharbitic acid were dissolved in distilled water at 100 mg/mL, respectively, and phar-genin was prepared in dimethyl sulfoxide at 10 mg/mL. Acetylcholine (ACh) chloride and atropine sulfate 1 mg/mL were prepared with distilled water.

### Statistical analysis

Results were expressed as means ± S.E.M. (*n* = 3 ~ 8). Statistical comparisons between two groups were carried out using a Student’s t-test. Multiple comparisons were performed using Dunnet-test. *: *p* < 0.05, **: *p* < 0.01, significantly different from the control.

## Results

### Effects of pharbitin, phar-genin and pharbitic acid on peristalsis of mouse colon

**Fig. 2:**
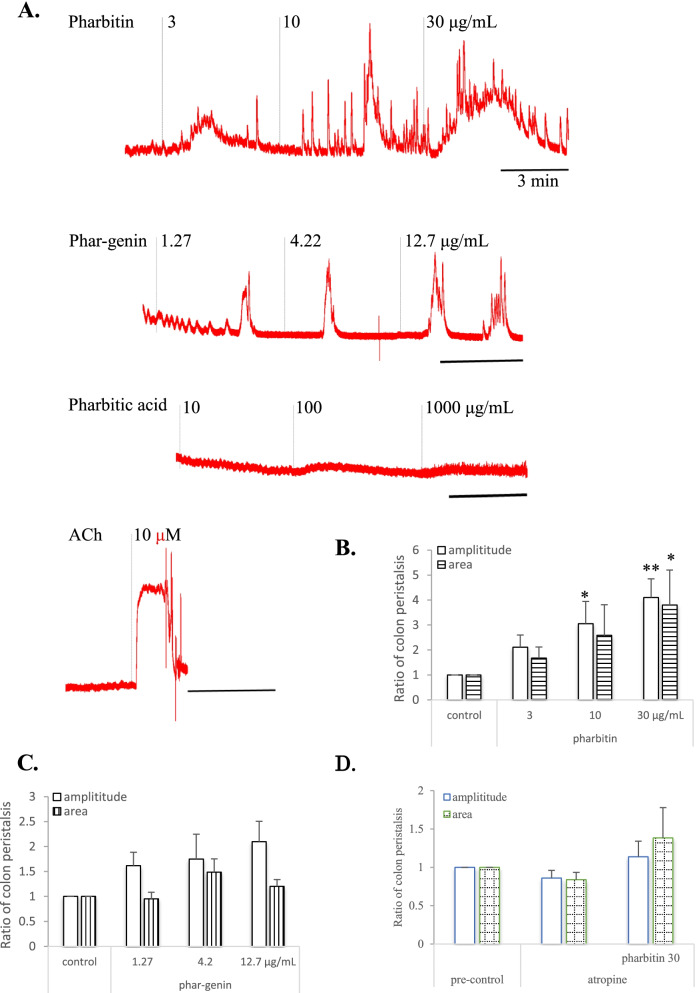
Effects of resin glycoside and its components on tonus of colon isolated from mice. **A**, representative record showing effect of pharbitin, phar-genin, pharbitic acid and acetylcholine (ACh) on tonus of colon. The grey vertical line shows the start of sample administration. **B**, normalized amplitude and area of colon tonus in the presence of pharbitin. **C**, normalized amplitude and area of colon tonus in the presence of phar-genin. Amplitude and area of colon tonus in the presence of pharbitin or phar-genin were normalized to mean amplitude and area recorded in pre-control (resting tonus), respectively. **D**, Normalized amplitude and area of colon tonus in the absence or presence of pharbitin and atropine. At first, colon tonus in Tyrode’s solution was recorded for 5 min as a pre-control. After stable, colon ring was pretreated with atropine of 10 μM for 3 min, and then treated with pharbitin of 30 μg/mL for 5 min

In mouse isolated colon, both pharbitin and phar-genin induced large tonic contraction, which is comparable to that by ACh 10 μM, and pharbitic acid-induced contraction was weak (Fig. 2A). Pharbitin at 3, 10 and 30 μg/mL concentration-dependently increased amplitudes of peristalsis by 2.11, 3.05 and 4.1fold, and intensities (areas) of peristalsis by 1.67, 2.58 and 3.8-fold, respectively (Fig. 2B). Phar-genin at 1.27, 4.2 and 12.7 μg/mL concentration-dependently increased amplitudes of peristalsis by 1.61, 1.75 and 2.1-fold, and had a trend to increase intensities of peristalsis by 0.95, 1.49 and 1.2-fold, respectively (Fig. 2C).

To investigate the possible mechanism of pharbitin-induced increment of colon tonus, we evaluated the effect of muscarinic receptor antagonist on colon tonus. As shown in Fig. 2D, pre-treatment with atropine, compared with the control, amplitude and area of spontaneous peristalsis were inhibited to 0.86 and 0.83-fold, and a little increased by pharbitin to 1.13 and 1.38-fold under atropine.

### Effects of pharbitin and phar-genin on peristalsis of mouse ileum

**Fig. 3:**
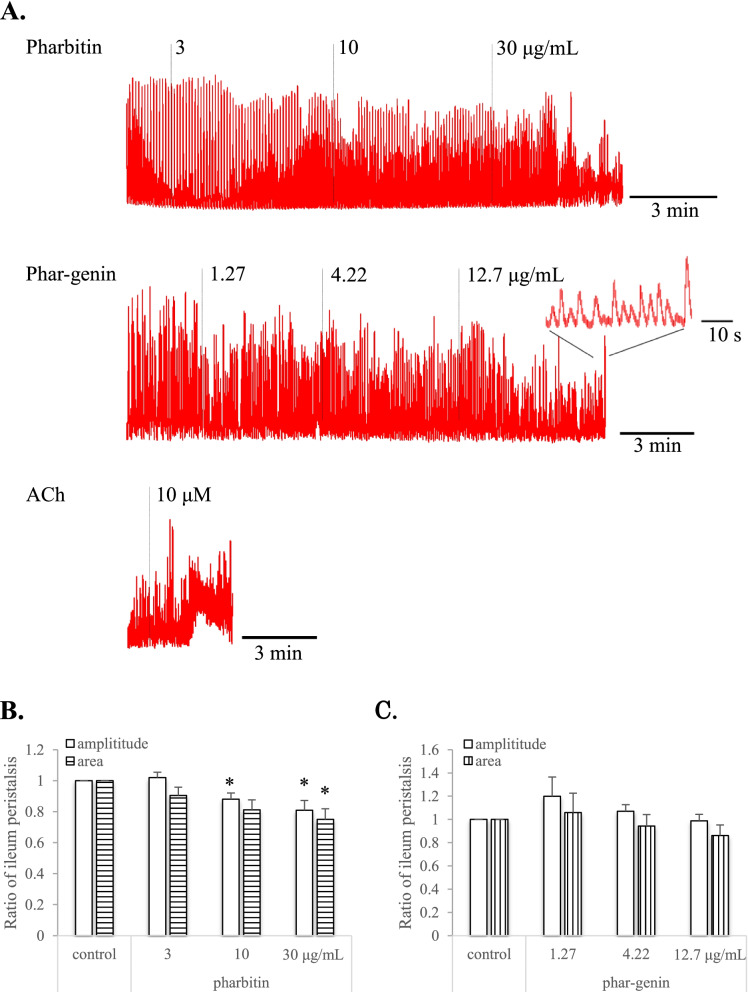
Effects of resin glycoside and its components on tonus of ileum isolated from mice. **A**, representative record showing effect of pharbitin, phar-genin and acetylcholine (ACh) on tonus of ileum. A partial enlarged view showing ileum peristalsis. **B**, normalized amplitude and area of ileum tonus in the presence of pharbitin. **C**, normalized amplitude and area of ileum tonus in the presence of phar-genin. Amplitude and area of ileum tonus in the presence of pharbitin or phar-genin were normalized to mean amplitude and area recorded in pre-control (resting tonus), respectively

In ileum (Fig. 3A), pharbitin and phar-genin had decreasing tendency on phasic contraction of ileum. Pharbitin at 3, 10 and 30 μg/mL concentration-dependently decreased amplitudes of peristalsis to 1.02, 0.88 and 0.81-fold, and intensities (areas) of peristalsis to 0.90, 0.81 and 0.75-fold, respectively (Fig. 3B). Phar-genin at 1.27, 4.2 and 12.7 μg/mL had amplitudes of peristalsis by 1.19, 1.07 and 0.99-fold, and had a trend to decrease intensities of peristalsis by 1.06, 0.94 and 0.86-fold, respectively (Fig. 3C). In the application of ACh, its 10 μM induced small tonic contraction, and phasic contraction was decreased at the same time (Fig. 3A).

## Discussion

Here, it is reported for the first time that the effects by resin glycoside of Pharbitidis Semen on colon tonus are different with those on ileum tonus isolated from mice.

Considering the complex of resin glycoside, at first, alkaline hydrolysis of pharbitin gave pharbitic acid, and acidic hydrolysis of pharbitic acid gave phar-genin, then their direct effects on mice isolated colon or ileum tension were investigated with Magnus method of ex vivo. It has been reported that the oral administration of pharbitin at 31.25–125 mg/kg induced diarrhea in rats, and pharbitin at 2–8 μg/mL decreased the expression of Aquaporin 3 in HT-29 cells [12]. Considering these in vivo and in vitro effects related to intestine motility, the possible concentrations of resin glycoside were chosen to be tested in present ex vivo. As shown in Fig. 2, pharbitin (3–30 μg/mL) and phar-genin (1.27–12.7 μg/mL) were found to increase both amplitudes and intensities (areas) of peristalsis on colon with a concentration-dependent manner. However, pharbitic acid (10–1000 μg/mL), as components of alkaline hydrolysis products of pharbitin, which was composed of four glycosidic acids even as acidic glycoside [1], evoked only weak peristalsis. The relation between chemical structure and activity remain to be investigated.

Then, in the presence of atropine (Fig. 2D), a muscarinic ACh receptor antagonist, amplitude and area of colon tension were increased to 4.1 and 3.8-fold by pharbitin alone at 30 μg/mL (Fig. 2B), and only 1.1 and 1.4-fold with pharbitin and atropine (Fig. 2D). It suggests that the increment of colon tension due to pharbitin was decreased by atropine. In the next step it is necessary to investigate details of its pharmacological mechanism ex vivo / in vivo. Pharmacological mechanism.

On the other hand, in ileum (Fig. 3), phasic contraction was decreased by pharbitin at 3, 10, 30 μg/mL, but pharbitin at same concentrations increased peristalsis on colon. In the case of phar-genin, seems no significant effect on ileum tonus, although induced peristalsis of colon. Thus, the selective contraction on colon by pharbitin and its components could be a basis for its purgative action.

## Conclusions

Pharbitin, active ingredient of Pharbitidis Semen, increased tonus of colon isolated from mice probably via ACh receptors, but decreased ileum tonus. Pharbitic acid evoked weak peristalsis. The selective contraction on colon by pharbitin and its components could be a basis for its purgative action.

## Data Availability

No additional information is supplied as a supplementary file. Additional questions or information may be obtained by contact the Corresponding author, Jian-Rong Zhou.

## Abbreviations

ACh: Acetylcholine
phar-genin: Aglycone fraction
